# A feature optimization study based on a diabetes risk questionnaire

**DOI:** 10.3389/fpubh.2024.1328353

**Published:** 2024-02-23

**Authors:** Liangjun Jiang, Zerui Yang, Gang Liu, Zhenhua Xia, Guangyao Yang, Haimei Gong, Jing Wang, Lei Wang

**Affiliations:** ^1^College of Information and Communication Engineering, State Key Lab of Marine Resource Utilization in South China Sea, Hainan University, Haikou, China; ^2^School of Electronics and Information, Yangtze University, Jingzhou, China; ^3^Shenzhen Center for Disease Control and Prevention, Shenzhen, China; ^4^E-link Wisdom Co., Ltd., Shenzhen, China

**Keywords:** diabetes, risk prediction, machine learning, feature enumeration, diabetes risk questionnaire, public health

## Abstract

**Introduction:**

The prevalence of diabetes, a common chronic disease, has shown a gradual increase, posing substantial burdens on both society and individuals. In order to enhance the effectiveness of diabetes risk prediction questionnaires, optimize the selection of characteristic variables, and raise awareness of diabetes risk among residents, this study utilizes survey data obtained from the risk factor monitoring system of the Centers for Disease Control and Prevention in the United States.

**Methods:**

Following univariate analysis and meticulous screening, a more refined dataset was constructed. This dataset underwent preprocessing steps, including data distribution standardization, the application of the Synthetic Minority Oversampling Technique (SMOTE) in combination with the Round function for equilibration, and data standardization. Subsequently, machine learning (ML) techniques were employed, utilizing enumerated feature variables to evaluate the strength of the correlation among diabetes risk factors.

**Results:**

The research findings effectively delineated the ranking of characteristic variables that significantly influence the risk of diabetes. Obesity emerges as the most impactful factor, overshadowing other risk factors. Additionally, psychological factors, advanced age, high cholesterol, high blood pressure, alcohol abuse, coronary heart disease or myocardial infarction, mobility difficulties, and low family income exhibit correlations with diabetes risk to varying degrees.

**Discussion:**

The experimental data in this study illustrate that, while maintaining comparable accuracy, optimization of questionnaire variables and the number of questions can significantly enhance efficiency for subsequent follow-up and precise diabetes prevention. Moreover, the research methods employed in this study offer valuable insights into studying the risk correlation of other diseases, while the research results contribute to heightened societal awareness of populations at elevated risk of diabetes.

## 1 Introduction

Diabetes is a prevalent chronic disease in modern society, which poses a serious threat to human health and daily life. Medical statistical and analytical studies have revealed that diabetes (including type 1 and type 2 diabetes) increases the risk of several complications, such as cardiovascular disease (1), heart failure (2), depression (3), non-alcoholic chronic fatty liver disease (4), cognitive decline (5), and functional impairment (5). These complications continue to impose a significant burden on millions of people with diabetes (5). Based on estimates from National Health and Nutrition Examination Survey data, the prevalence of diabetes among adults in the United States increased significantly between 1999–2000 and 2017–2018 (6). The prevalence of diabetes in China increased from <1% in the 1980s to nearly 11% in 2013 and continued to rise from 2013 to 2018 (7). According to big data statistics and inferences from the International Diabetes Federation (IDF), the global prevalence of diabetes among the age group of 20–79 is estimated to be 10.5% (536.6 million people) in 2021, and it will rise to 12.2% (783.2 million people) in 2045, meaning that over 10.5% of adults worldwide have diabetes (8). Especially in the era of the Corona Virus Disease 2019 (COVID-19) pandemic, COVID-19 patients have shown an increased risk and burden of diabetes (9–11), and COVID-19 patients who have diabetes themselves will have a significantly increased risk of hospitalization and death (12). Based on these realities, more measures are needed to increase the public's awareness of the hazards of diabetes and enhance the ability to predict the risk of diabetes.

With the significant improvement in electronic computing power, the field of artificial intelligence has also ushered in a wave of development. Machine learning (ML) is a constantly evolving branch of computer algorithms and the core of artificial intelligence. Its purpose is to simulate human intelligence by learning from the surrounding environment (13). Its emergence has brought about interdisciplinary applications in many fields. With the increasing attention to “big data,” it has provided epidemiologists with new tools to solve problems that are not suitable for classical methods (14). Therefore, in recent years, many ML models have been proposed for the risk prediction of diabetes. Xu et al. collected 15,166 health examination data from eastern China and constructed a multifactorial regression model (LASSO regression and logistic regression) to predict the risk of type 2 diabetes. The statistical accuracy of the model was evaluated using the area under the ROC curve (AUC), which reached 0.865, indicating good predictive performance (15). Gollapalli et al. (16) used a dataset from a hospital in Saudi Arabia, which included 897 hospitalized patients with 10 unique features. Among them, 731 patients had prediabetes, 89 patients were diagnosed with type 1 diabetes, and 77 patients had type 2 diabetes. By conducting multiple experiments using support vector machine (SVM), random forest, K-nearest neighbor (KNN), decision tree, bagging, and stacking algorithms, a new stacking model combining bagging KNN, bagging decision tree, and KNN classifiers showed good performance, with KNN classifier accuracy, weighted recall, weighted precision, and kappa score of 94.48%, 94.48%, 94.70%, and 0.9172, respectively (16). Dritsas et al. (17) conducted their experiment based on patient data from the Sylhet Diabetes Hospital in Sylhet, Bangladesh. This dataset^1^ was collected through direct questionnaires and diagnostic results. In this study, various ML models were evaluated based on the metrics of precision, recall, FMeasure, accuracy, and AUC. The models were compared using 10-fold cross-validation and data splitting, and the final results showed that random forest and KNN were the best-performing models (17). Currently, the most widely used dataset is the publicly available Pima Indian dataset, which includes 768 data results with nine feature variables. Many studies (18–26) have established diabetes risk prediction models based on this dataset, mostly using classical ML algorithms such as logistic regression, random forest, SVM, decision tree, KNN, gradient boosting, naive bayes, and neural networks. These studies provide some direction for the selection of models in this article.

This study identified several issues in the research of diabetes risk prediction. First, most of the feature variables used in many diabetes risk prediction models are patient physical examination data or various laboratory testing indicators in blood components (such as blood pressure, creatinine, triglycerides, etc.). These feature indicators require professional testing in hospitals, thus the risk models built based on these features are not conducive to widespread application in the population. Second, the training dataset still suffers from the problem of a small number of samples. The widely used Pima Indian dataset only includes data from 768 patients, and the small sample size makes it difficult to validate the model's generalizability in the population. Third, the model construction process lacks relevant correlation studies on risk factors for diabetes, and the feature variables used in the dataset lack clear analysis and comparison.

In response to the above-mentioned issues, this study believes that constructing a questionnaire-based dataset is more conducive to statistical analysis of diabetes risk factors and enhancing residents' awareness of diabetes risk. Therefore, this study conducted experiments based on the 2021 behavior risk factor monitoring system (BRFSS) data from the United States. The feature variables of this dataset are more concerned about the disease conditions and behavioral factors of the crowd. After performing a univariate analysis of the original data, this study selected the diabetes risk factor-related feature variables and extracted them to form a new dataset, which included 228,697 data samples with 21 feature variables per data sample. After conducting a numerical correlation analysis on the variables in the new dataset, we can preliminarily observe the impact of each factor on diabetes. The preprocessed dataset was used to build models using multiple ML algorithms, and their performance was compared to select the optimal ML algorithm. Then, by enumerating the feature variables, this study verified the impact of various risk factors on the risk of developing diabetes to optimize the number of variables in the diabetes risk prediction questionnaire.

## 2 Materials and methods

### 2.1 Original dataset background and analysis

**Figure 1:**
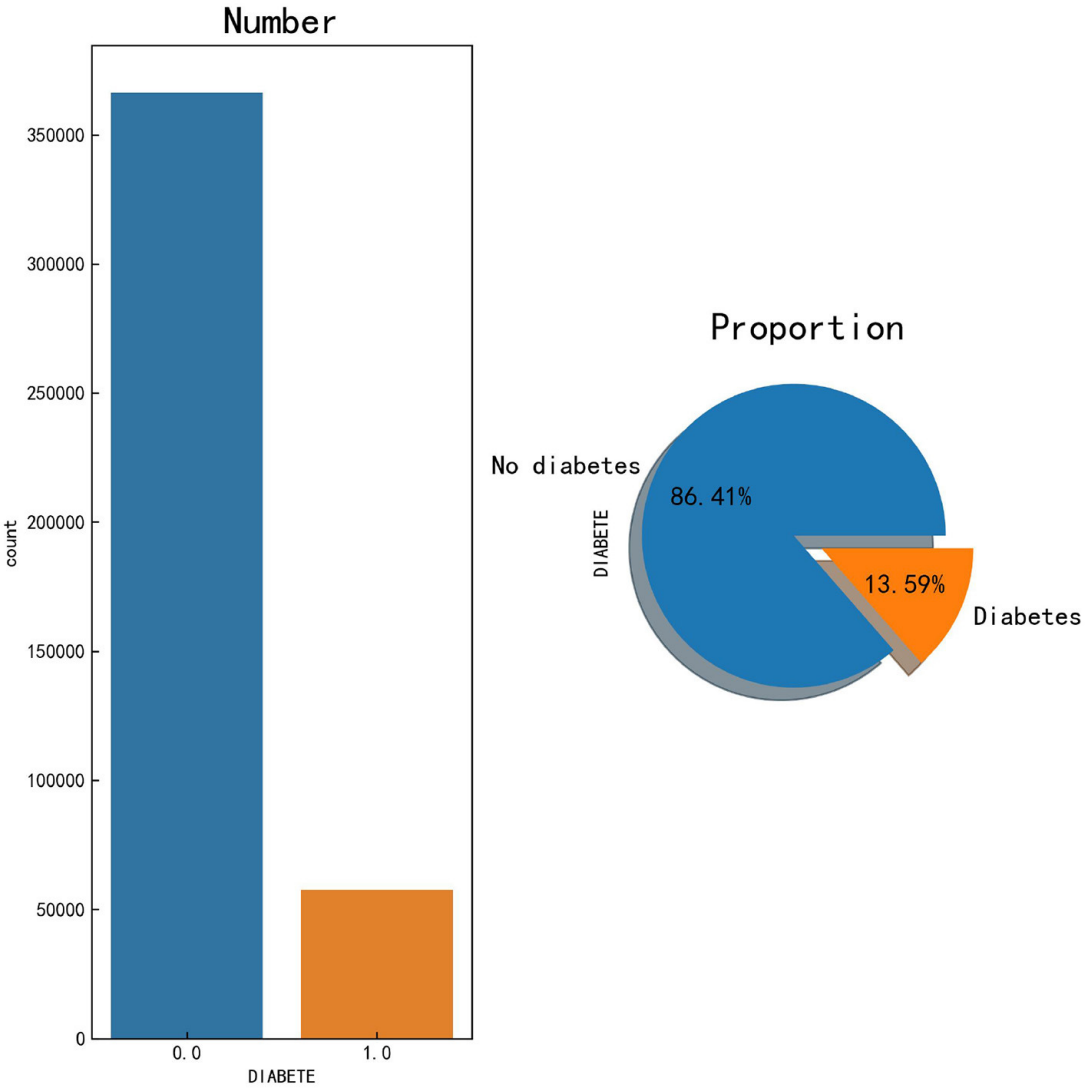
Number and proportion of participants with and without diabetes.

To identify risk factors for various human diseases, the Centers for Disease Control and Prevention in the United States launched the Behavioral Risk Factor Surveillance System (BRFSS) in 1984, which is a survey conducted using random landline and mobile phone numbers, targeting adult populations in all US states (27). The BRFSS collects data on risk behaviors and preventive health practices that may affect participants' health status. The dataset used in this study was obtained from the 2021 BRFSS survey,^2^ which includes 303 feature variables and encompasses 438,693 participants. From these raw data, we summarized the number of participants with and without diabetes, as shown in Figure 1. Based on the compiled data, the figure shows that the prevalence of diabetes among the participants is 13.59%, which is higher than the estimated global prevalence of 10.5% by the IDF. This indicates that we should further investigate the related risk factors for diabetes to improve risk awareness and enhance the predictive capability of big data ML models for high-risk populations.

In this study, we classified and counted all the feature variables in the original dataset, as shown in Table 1. The table clearly shows that the BRFSS survey data covers a wide range of issues related to human daily life, which is highly relevant to the objectives of this study.

**Table 1:** 2021 BRFSS survey data classification statistics.

**Classification name**	**Representative characteristic factors**	**Number of features**
Questionnaire information	The state in which the collection belongs, Date, Whether the access is complete, Serial number, Questionnaire version	28
Basic personal information	Sex, Age, Education, Marriage, Weight, Height	46
Family situation	Number of adults, parental relationship, accommodation relationship, family children's situation	34
Illness	High blood pressure, heart disease, kidney disease, cancer, depression, stroke	72
Medical testing and surgery	Frequency of medical examinations, uterine surgery, stool tests, eye tests, medical services	34
Self-health assessment	Mental health evaluation, physical health evaluation, physical condition in the past 30 days	5
Eating behaviors	Vegetables, fruits, juices, smoking, drinking, salt	47
Problems with daily activities	Frequency of exercise, difficulty walking or climbing stairs, difficulty concentrating	9
Identity and income	Identity, property, income level	4
Other	Military service, sexual contact, firearms, pregnancy, insurance	24

**Figure 2:**
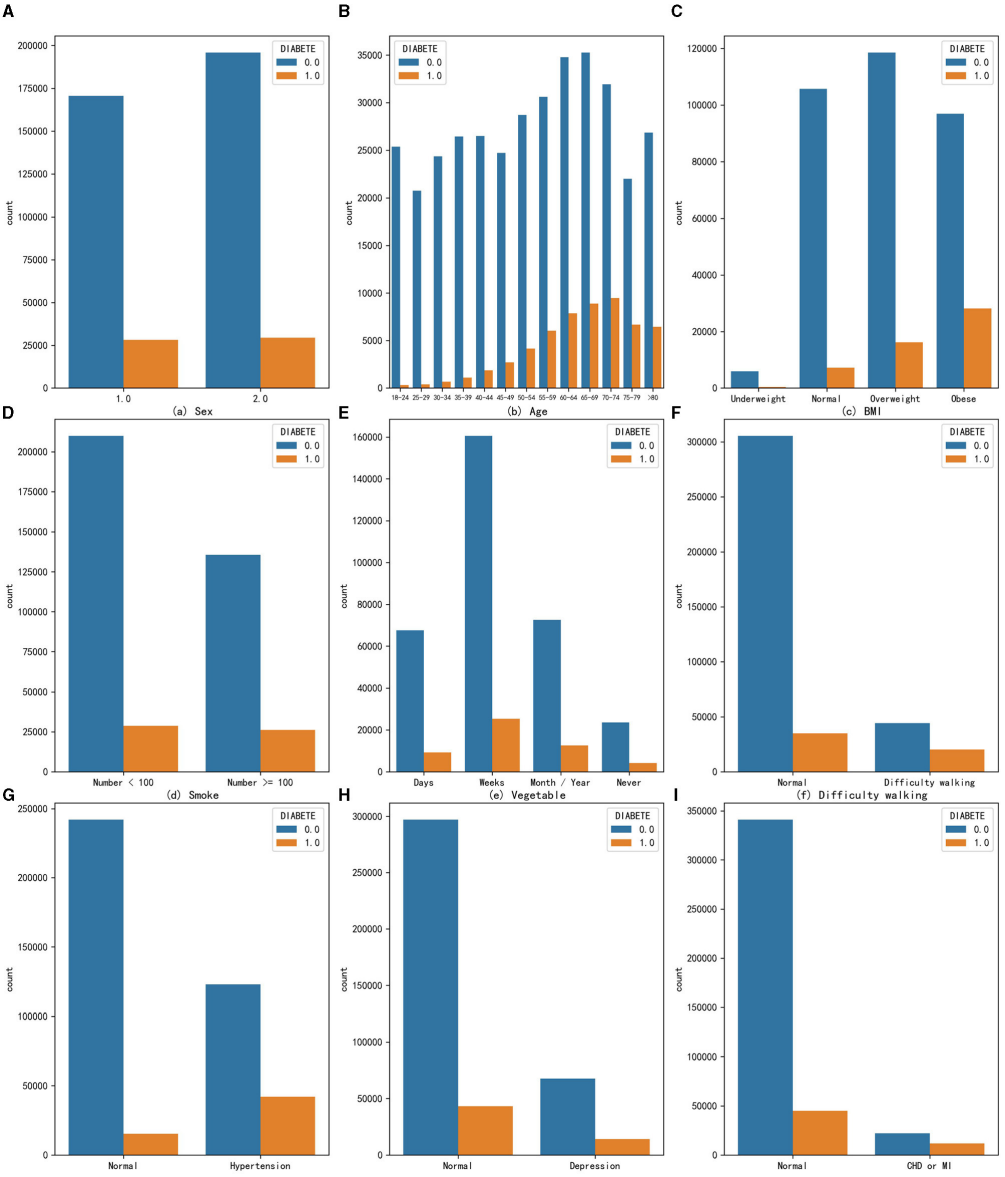
The distribution of people with and without diabetes across nine characteristic variables **(A)** Sex, **(B)** Age, **(C)** BMI, **(D)** Smoke, **(E)** Vegetable, **(F)** Difficulty walking, **(G)** Hypertension, **(H)** Depression, and **(I)** CHD or MI.

Although genetic structure may determine to some extent an individual's response to environmental changes, medical research has shown that excessive accumulation of fat (28) and alcohol addiction (29) can increase the risk of diabetes. In addition, according to the IDF list, important risk factors for diabetes include family history of diabetes, overweight, unhealthy diet, lack of exercise, aging, hypertension, ethnicity, and impaired glucose tolerance (20). In this study, we conducted univariate analysis of some common risk factors for diabetes on this dataset, combining these research conclusions. Finally, 9 variables were found that have a certain impact on the proportion of diabetic people. The statistical results of their quantitative distribution are shown in Figure 2. The figure shows the distribution people with and without diabetes across nine characteristic variables, so the proportion of diabetic patients can be analyzed in different variables, where a value of 0 means no diabetes and a value of 1 means diabetes. The analysis of different feature variables is as follows:

Sex: The prevalence of diabetes in the male population is 14.19%, while in the female population it is 13.06%. From the perspective of univariate effects, sex has a slight impact on the probability of developing diabetes, with men being more likely to develop diabetes.

Age: _AGEG5YR variable in the original dataset divides the age of adults over 18 into 13 intervals, each interval being approximately 5 years. As age increases, the number of people with diabetes gradually increases, and the proportion of people with diabetes also increases, indicating that age is a risk factor for diabetes and that the risk of developing diabetes increases with age. The highest proportion of diabetes occurs in the age group of 75 to 79 years, reaching 23.33%.

Obesity: Body mass index (BMI) is a standard for measuring the degree of obesity in the human body, which is calculated by dividing weight by height squared. The _BMI5CAT variable in the original dataset classifies BMI values into underweight, normal weight, overweight, and obese categories based on the standards of thinness and obesity. From the graph, it can be seen that obesity increases the number of people with diabetes in the population, and the proportion of diabetes also increases, with the proportion of diabetes reaching 22.55% in the obese population, indicating that the obese population needs to pay more attention to the risk of diabetes.

Excessive smoking: According to calculations, the proportion of people with diabetes in the population who smoke more than 100 cigarettes is 4.1% higher than those who smoke <100 cigarettes, suggesting that smoking is also a possible risk factor for diabetes.

Habit of eating vegetables: The original dataset variable FVGREEN1 records the frequency of eating green vegetables. The graph shows four categories: daily, weekly, monthly or yearly, and not eating. After calculating the proportion of diabetes, it was found that the higher the frequency of eating green leafy vegetables, the lower the proportion of diabetes, indicating that eating more green leafy vegetables can reduce the risk of developing diabetes to a certain extent.

Difficulty walking: The data indicate that the proportion of diagnosed diabetes is significantly higher among populations with difficulty walking or climbing stairs, reaching 36.53%, about 24 percentage points higher than the people without such problems. Therefore, the population with difficulty walking or climbing stairs should also be closely monitored for the presence of diabetes.

High blood pressure: The data suggest that the risk of developing diabetes is particularly high among those with hypertension. In this group, the proportion of individuals with diabetes is as high as 25.49%.

Depression: Individuals with depression or other mental disorders are also at an increased risk of developing diabetes, with a disease prevalence of 17.25%, which is 4.55% higher than that in the people without such problems. Depression is a comorbidity of diabetes, and individuals with depression or other mental health issues should pay special attention to their diabetes risk.

Coronary heart disease (CHD) or myocardial infarction (MI): The _MICHD variable in the original dataset recorded the population with coronary heart disease or myocardial infarction. Both diseases are types of cardiovascular diseases and are also complications of diabetes. The statistics show that the proportion of diabetes in this population is as high as 34.62%, meaning that one-third of people with these diseases also have diabetes. This proportion is much higher than the prevalence of diabetes in the people without such problems. Therefore, coronary heart disease or myocardial infarction is a very high-risk factor for diabetes.

### 2.2 Feature variable extraction

To explore the comprehensive impact of various risk factors on diabetes using ML, this study needs to extract multiple feature variables from a large original data set to construct a new dataset. First, based on the results of univariate analysis of the original data set, we considered which variables were highly relevant and significant in diabetes risk assessment. For example, factors such as high blood pressure, high cholesterol, smoking, and obesity are widely recognized as major risk factors for diabetes. Second, we focused on variables that reflect lifestyle and behavioral habits, such as dietary habits (fruit and vegetable intake) and physical activity, which are key factors affecting individual health status. Additionally, we considered socioeconomic factors such as education and income level, as these factors have been shown to have a significant impact on health status. Finally, considering the sample size requirements, features with effective data volume of feature variables >200,000 in the original data set were selected. Our choices are intended to create a comprehensive model that accurately reflects an individual's health status and diabetes risk. This study finally selected 21 feature variables to construct a new data set. The names of the feature variables, their meanings, and the explanations in the original dataset are shown in Table 2. This study also extracted the healthy population and diabetes patients from the DIABETE4 variable in the original dataset and used them as labels for the dataset. The value 0 represents a person who has not had diabetes, and the value 1 represents a person who has had diabetes.

**Table 2:** Filtered characteristic variable description.

**Serial number**	**Variable original name**	**Replaced name**	**Characteristic variable meaning**	**Characteristic variable problem statement**
1	_RFHYPE6	HighBP	High blood pressure	Question: adults who have been told they have high blood pressure by a doctor, nurse, or other health professional. Value: 1: No high blood pressure; 2: Have high blood pressure; 9: Don't know/Not Sure/Refused/Missing.
2	TOLDHI3	HighChol	High cholesterol	Question: Adults who have had their cholesterol checked and have been told by a doctor, nurse, or other health professional that it was high. Value: 1: No high cholesterol; 2: High cholesterol; 9: Don't know/Not Sure/Refused/Missing.
3	_CHOLCH3	CholCheck	Cholesterol checking habits	Question: Cholesterol check within past five years. Value: 1: Had cholesterol checked in past 5 years; 2: Did not have cholesterol checked in past 5 years; 3: Have never had cholesterol checked; 9: Don't know/Not Sure or Refused/Missing.
4	_BMI5	BMI	Fat and thin degree	Question: Body Mass Index (BMI).
5	SMOKE100	Smoker	Smoking	Question: Have you smoked at least 100 cigarettes in your entire life? [Note: 5 packs = 100 cigarettes] Value: 1: Have smoked more than 100 cigarettes; 2: Have smoked less than 100 cigarettes; 7: Don't know/Not Sure; 9: Refused.
6	CVDSTRK3	Stroke	Stroke	Question: (Ever told) (you had) a stroke. Value: 1: Had a stroke; 2: Never had a stroke; 7: Don't know/Not sure; 9: Refused.
7	_MICHD	Heartproblems	Coronary heart disease or myocardial infarction	Question: Respondents that have ever reported having coronary heart disease (CHD) or myocardial infarction (MI). Value: 1: Reported having MI or CHD; 2: Did not report having MI or CHD.
8	_TOTINDA	PhysActivity	Physical activity and exercise	Question: Adults who reported doing physical activity or exercise during the past 30 days other than their regular job. Value: 1: Had physical activity or exercise; 2: No physical activity or exercise in last 30 days; 9: Don't know/Refused/Missing.
9	_FRTLT1A	Fruits	Fruit diet	Question: Consume Fruit 1 or more times per day. Value: 1: Consumed fruit one or more times per day; 2: Consumed fruit < one time per day; 9: Don't know, refused or missing values.
10	_VEGLT1A	Veggies	Vegetable diet	Question: Consume Vegetables 1 or more times per day. Value: 1: Consumed vegetables one or more times per day; 2: Consumed vegetables < one time per day; 9: Don't know, refused or missing values.
11	_DRNKWK1	AlcoholConsump	Drinking	Question: calculated total number of alcoholic beverages consumed per week.
12	_HLTHPLN	AnyHealthcare	Health insurance	Question: adults who had some form of health insurance. Value:1: have some form of insurance; 2: Do not have some form of health insurance; 9: Don't know, refused or missing insurance response.
13	MEDCOST1	NoDocbcCost	No money is to cover visiting doctor costs	Question: Was there a time in the past 12 months when you needed to see a doctor but could not because you could not afford it? Value: 1: Yes; 2: No; 7: Don't know/Not sure; 9: Refused.
14	GENHLTH	GenHlth	Self-evaluation of general health	Question: Would you say that in general your health is: Value: 1: Excellent; 2: Very good; 3: Good; 4: Fair; 5: Poor; 7: Don't know/Not Sure; 9: Refused
15	MENTHLTH	MentHlth	Mental health	Question: Now thinking about your mental health, which includes stress, depression, and problems with emotions, for how many days during the past 30 days was your mental health not good? Value: 1–30: Number of days; 88: None; 77: Don't know/Not Sure; 99: Refused
16	PHYSHLTH	PhysHlth	Physical health	Question: Now thinking about your physical health, which includes physical illness and injury, for how many days during the past 30 days was your physical health not good? Value: 1-30: Number of days; 88: None; 77: Don't know/Not Sure; 99: Refused
17	DIFFWALK	DiffWalk	Difficulty in action	Question: Do you have serious difficulty walking or climbing stairs? Value: 1: Yes; 2: No; 7: Don't know/Not sure; 9: Refused
18	_SEX	Sex	Sex	Question: Calculated sex variable. Value:1: Male; 2: Female.
19	_AGEG5YR	Age	Age rating	Question: Fourteen-level age category. The last level indicates a minor
20	EDUCA	Education	Educational attainment	Question: What is the highest grade or year of school you completed? Value: 1: Never attended school or only kindergarten; 2: Elementary; 3: Some high school; 4: High school graduate; 5: Some college or technical school; 6: College graduate; 9: Refused.
21	INCOME3	Income	Household income	Question: Annual household income Value: Income is divided into 11 levels.

We also conducted statistical analysis in multiple dimensions (mean, standard deviation, *P* value, etc.) on the data set composed of 21 variables, as shown in Table 3. Based on the statistical analysis and *P* values provided, we can see that specific variables have a significant association with the incidence of diabetes.

**Table 3:** Statistics of each characteristic variable of the new dataset.

**Feature variable**	**mean**	**std**	**min**	**25%**	**50%**	**75%**	**max**	***P*-Value**
Diabetes	0.15	0.35	0	0	0	0	1	/
HighBP	0.42	0.49	0	0	0	1	1	0
HighChol	0.40	0.49	0	0	0	1	1	0
CholCheck	0.96	0.19	0	1	1	1	1	1.4295E-250
BMI	28.88	6.51	12	24	28	32	99	0
Smoker	0.41	0.49	0	0	0	1	1	3.1136E-173
Stroke	0.04	0.19	0	0	0	0	1	0
Heartproblems	0.09	0.28	0	0	0	0	1	0
PhysActivity	0.22	0.41	0	0	0	0	1	0
Fruits	0.38	0.48	0	0	0	1	1	9.42128E-46
Veggies	0.17	0.38	0	0	0	0	1	2.0393E-113
AlcoholConsump	298.52	825.44	0	0	23	300	53,200	2.8686E-228
AnyHealthcare	0.04	0.19	0	0	0	0	1	4.25319E-40
NoDocbcCost	0.06	0.24	0	0	0	0	1	3.25676E-13
GenHlth	1.47	1.03	0	1	1	2	4	0
MentHlth	3.90	7.85	0	0	0	3	30	6.15104E-77
PhysHlth	3.72	8.22	0	0	0	2	30	0
DiffWalk	0.15	0.36	0	0	0	0	1	0
Sex	0.48	0.50	0	0	0	1	1	1.68085E-51
Age	7.86	3.24	1	5	8	10	13	0
Education	0.86	0.94	0	0	1	2	5	0
Income	4.06	2.37	0	2	4	6	10	0

### 2.3 Feature variable optimization

From the feature variable descriptions in Table 2, it can be seen that the original dataset's feature variable values are not consistently standardized. Therefore, this study has set uniform distribution standards for some variable values, starting from zero. In the case of disease variables, lower values, such as zero, indicate better health. The variable _AGEG5YR in the original dataset represents the 14th level age group, which includes minors or other cases that are not relevant to the objectives of this study. Therefore, data samples contained in this age group were excluded from the analysis. Additionally, data samples with missing or incomplete answers, such as “do not know” or “refuse to answer,” were removed from the dataset. Redundant samples with identical values for all feature variables and labels were also eliminated. As a result, a new dataset containing 225,998 data samples and 21 different feature variables was created. This dataset includes 192,486 non-diabetic individuals and 33,512 diabetic individuals, with a diabetic prevalence rate of 14.82%, which is similar to the initial large dataset's prevalence rate. Table 4 explains the meanings of the feature variables in the new dataset.

**Table 4:** Description of new dataset variable values.

**Serial number**	**Feature variable name**	**Characteristic variable value description**
1	HighBP	0: No high blood pressure; 1: High blood pressure.
2	HighChol	0: No high cholesterol; 1: High cholesterol.
3	CholCheck	0: Not checked; 1: Checked.
4	BMI	Weight/(height ^*^ height).
5	Smoker	0: The number of cigarettes smoked is < 100 sticks (5 boxes). 1: The number of cigarettes smoked is not < 100 sticks (5 boxes).
6	Stroke	0: No stroke; 1: Stroke.
7	Heartproblems	0: Normal; 1: Coronary heart disease or Myocardial infarction.
8	PhysActivity	0: Physical exercise; 1: No physical exercise.
9	Fruits	0: Consume fruit once or more times a day; 1: No daily consumption habit.
10	Veggies	0: Consume vegetables once or more times a day; 1: No daily vegetable habits.
11	AlcoholConsump	Total number of alcoholic beverages consumed per week.
12	AnyHealthcare	0: Participation in health insurance; 1: No health insurance.
13	NoDocbcCost	0: Can afford medical expenses; 1: Inability to afford medical expenses.
14	GenHlth	0: Excellent; 1: Very good; 2: Good; 3: Fair; 4: Poor.
15	MentHlth	Number of mentally unhealthy days in a month.
16	PhysHlth	Number of days of physical unhealthiness in a month.
17	DiffWalk	0: No difficulty walking or climbing stairs; 1: Severe difficulty walking or climbing stairs.
18	Sex	0: Female; 1: Male.
19	Age	The age categories are divided into 13 levels, with level 0 starting from 18 years old and level 12 representing people over 80 years old.
20	Education	0: College graduate; 1: Some college or technical school; 2: High school graduate; 3: Some high school; 4: Elementary; 5: Never attended school or only kindergarten.
21	Income	Annual household income level (level 0 is not < $200,000, level 10 is < $10,000).

From According to Table 4, it can be seen that the different feature variables represented in the new dataset have inconsistent units and real-world attributes. For example, the HighBP feature variable only includes two values, 0 and 1, while the BMI feature variable has a wide range of values. This difference in value magnitude between different feature variables can significantly affect the performance of the ML model. In order to improve the generalization performance of the classifier and the convergence speed of the algorithm, in the subsequent experiments, each feature dimension of the training set is standardized using Equation 1 by subtracting the mean and dividing by the standard deviation. For the test set, the standardization is performed using the mean and variance obtained from the training set.


(1) $$\begin{array}{l} \text{X}=\frac{\text{x}-\mu }{\sigma } \end{array}$$

In the equation, *X* represents the sample data of a certain feature column in the training set; μ represents the mean value of the sample data of the corresponding feature column of x; σ represents the standard deviation of the sample data of the corresponding feature column of x. By standardizing the data so that the mean of each feature dimension is 0 (as seen in Equation 2) and the standard deviation is 1 (as seen in Equation 3), the features of the dataset are made to conform to a standard normal distribution.


(2) $$\begin{array}{l} \mu^{*}=\frac{\sum \text{X}}{\text{n}}=\frac{\sum \text{x}-\sum \frac{\sum \text{x}}{\text{n}}}{\text{n}\sigma }=\frac{\sum \text{x}-\sum \text{x}}{\text{n}\sigma }=0 \end{array}$$

(3) $$\begin{array}{l} \sigma^{*}=\sqrt{\frac{\sum {\left(\text{X}-0\right)}^{2}}{\text{n}}}=\sqrt{\frac{\sum {\left(\text{x}-\mu \right)}^{2}}{\text{n}\sigma^{2}}}=\sqrt{\frac{\sum {\left(\text{x}-\mu \right)}^{2}}{\sum {\left(\text{x}-\mu \right)}^{2}}}=1 \end{array}$$

In the above equations: *X* represents the sample data of the standardized feature column; *n* represents the number of samples of the feature column.

### 2.4 Hardware and algorithm

This study utilized multiple ML algorithms to train the new dataset and construct models, in order to select the optimal ML algorithm for feature variable enumeration experiments. The computational efficiency of ML algorithms is related to both the hardware performance of the computer and the complexity of the algorithm. The hardware information of the computer equipment used in the experiments is as follows:

Central Processing Unit (CPU): 11th Gen Intel(R) Core (TM) i5-1135G7 @ 2.40 GHz.

Random Access Memory (RAM): Win32_PhysicalMemory 16G.

Solid State Drives (SSD): NVMe wDC PC SN530 SDBPNPZ-512G-1002.

The range of ML algorithms studied and compared in this article is relatively comprehensive, including the Gaussian naive Bayes (GaussianNB) algorithm suitable for data with gaussian distribution, logistic regression (LR) algorithm, SVM, KNN, classification and regression tree (CART) algorithm based on feature selection using the Gini index, bagging algorithm based on ensemble learning (30), random forest algorithm with decision tree as weak classifier (RF), gradient boosting decision tree (GBDT) algorithm (31), extreme gradient boosting (XGBoost) algorithm based on GBDT optimization (32), and light gradient boosting machine (LightGBM) algorithm based on XGBoost to improve operating efficiency (33). In this study, the dataset was split into training and testing sets with a ratio of 0.75:0.25, and the testing set consisted of 56,500 samples.

## 3 Results

### 3.1 Featured numerical correlation

**Figure 3:**
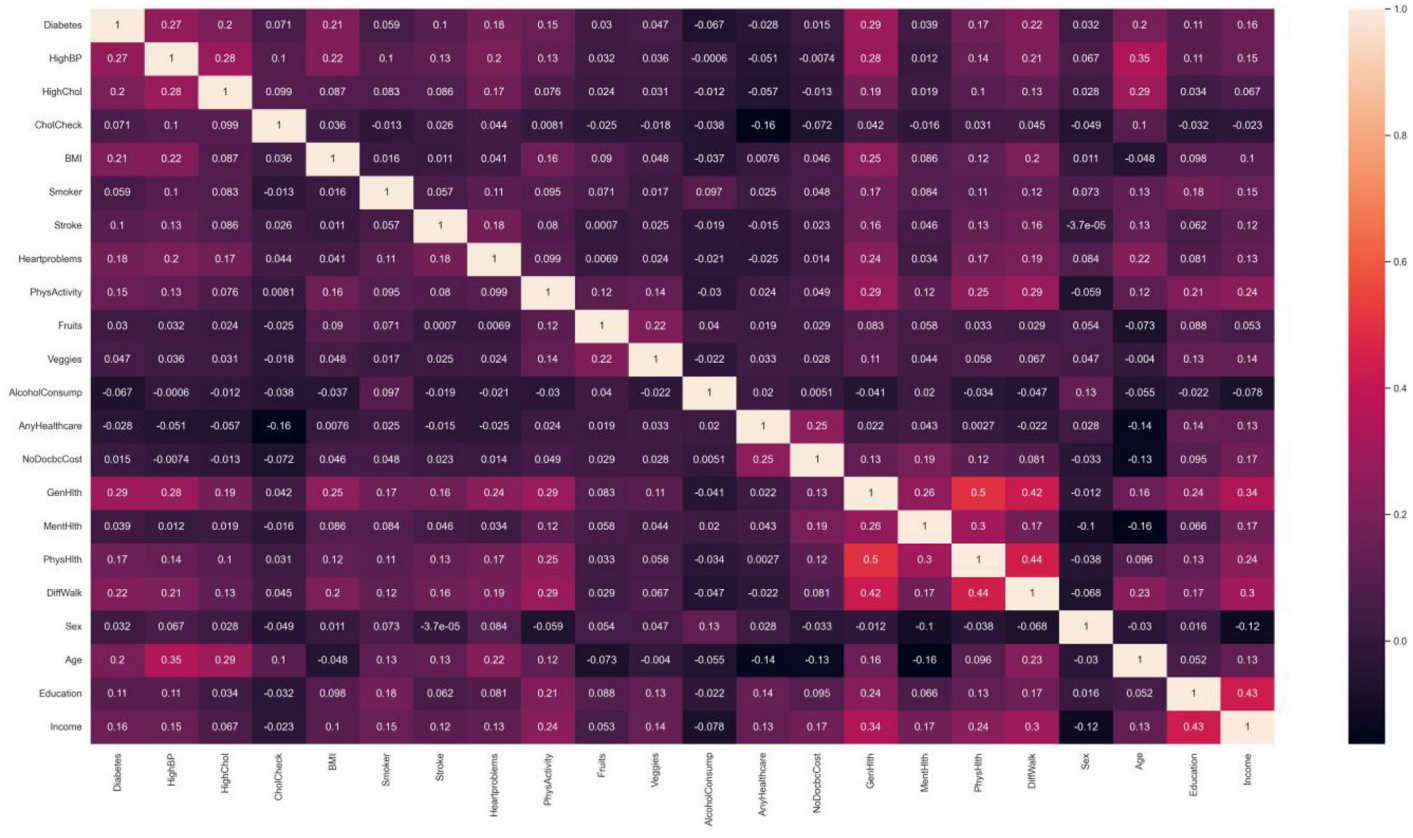
Related heat map of variable value. Factors that have shown strong correlation with diabetes include high blood pressure, high cholesterol, obesity, coronary heart disease or myocardial infarction, self-rated health, difficulty walking or climbing stairs, age, etc.

Numerical correlation analysis was conducted on the diabetes label variable and variables in the new dataset, and the resulting heat map of numerical correlation is shown in Figure 3. Based on the purpose of studying the risk factors for diabetes, this study focused more on the first row of data, namely the correlation between the numerical values of 21 feature variables and the numerical values of the diabetes label variable. It can be seen from the figure that factors with strong correlations include high blood pressure, high cholesterol, obesity, coronary heart disease or myocardial infarction, self-rated health status, difficulty walking or climbing stairs, and age. This indicates that from the perspective of numerical correlation analysis, age, obesity, and high blood pressure do indeed have a greater impact on the incidence of diabetes. In addition, income also has a certain degree of correlation, which indicates that diabetes prevention needs to pay more attention to low-income populations. To achieve the goal of simplifying the number of feature variables in the model more accurately, subsequent experiments will adopt the method of combining ML with feature variable enumeration to verify whether the prediction effect of diabetes risk after combining strongly correlated feature variables can approach that of the full variable prediction effect.

### 3.2 Algorithm comparison

**Figure 4:**
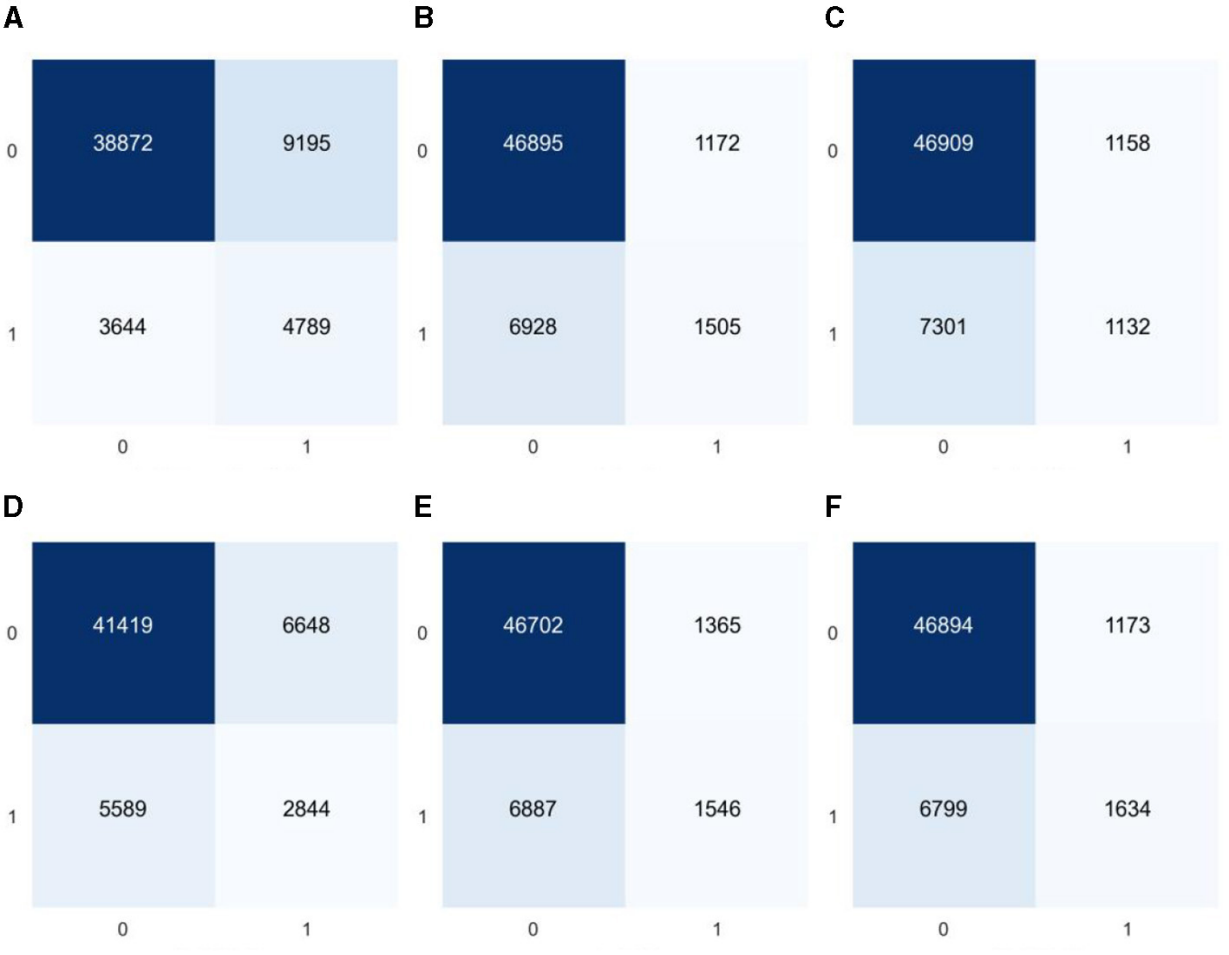
Classification confusion matrices for prediction results of six common machine learning models based on imbalanced datasets **(A)** GaussianNB, **(B)** LR, **(C)** KNN, **(D)** CART, **(E)** RF, and **(F)** GBDT.

As the model constructed from the dataset in this study is a binary classification model and the proportion of diabetes label samples in the dataset is imbalanced, it was found in the experiment that ML algorithms tend to predict the class with a higher proportion of labels (non-diabetic) more. For example, the classification confusion matrix of the models established by GaussianNB, LR, KNN, CART, RF, and GBDT after prediction is shown in Figure 4. It can be seen from the figure that the imbalanced dataset results in lower recall performance of the model in predicting the minority class samples (people with diabetes), indicating insufficient risk detection ability for the people with diabetes.

To improve the recall ability of the model for the people with diabetes, this study used the synthetic minority oversampling technique (SMOTE) to balance the number of samples corresponding to the dataset labels, thereby reducing the impact of sample imbalance on the recall performance of the people with diabetes in the ML model. The SMOTE algorithm uses the k-nearest neighbor approach to synthesize new samples by calculating the Euclidean distance between minority class samples. The feature variables of the new samples are calculated and generated using the following Equation 4.


(4) $$\begin{array}{l} {\text{x}}_{\text{n}}={\text{x}}_{\text{i}}+\zeta \left({\text{x}}_{\text{k}}-{\text{x}}_{\text{i}}\right) \end{array}$$

**Figure 5:**
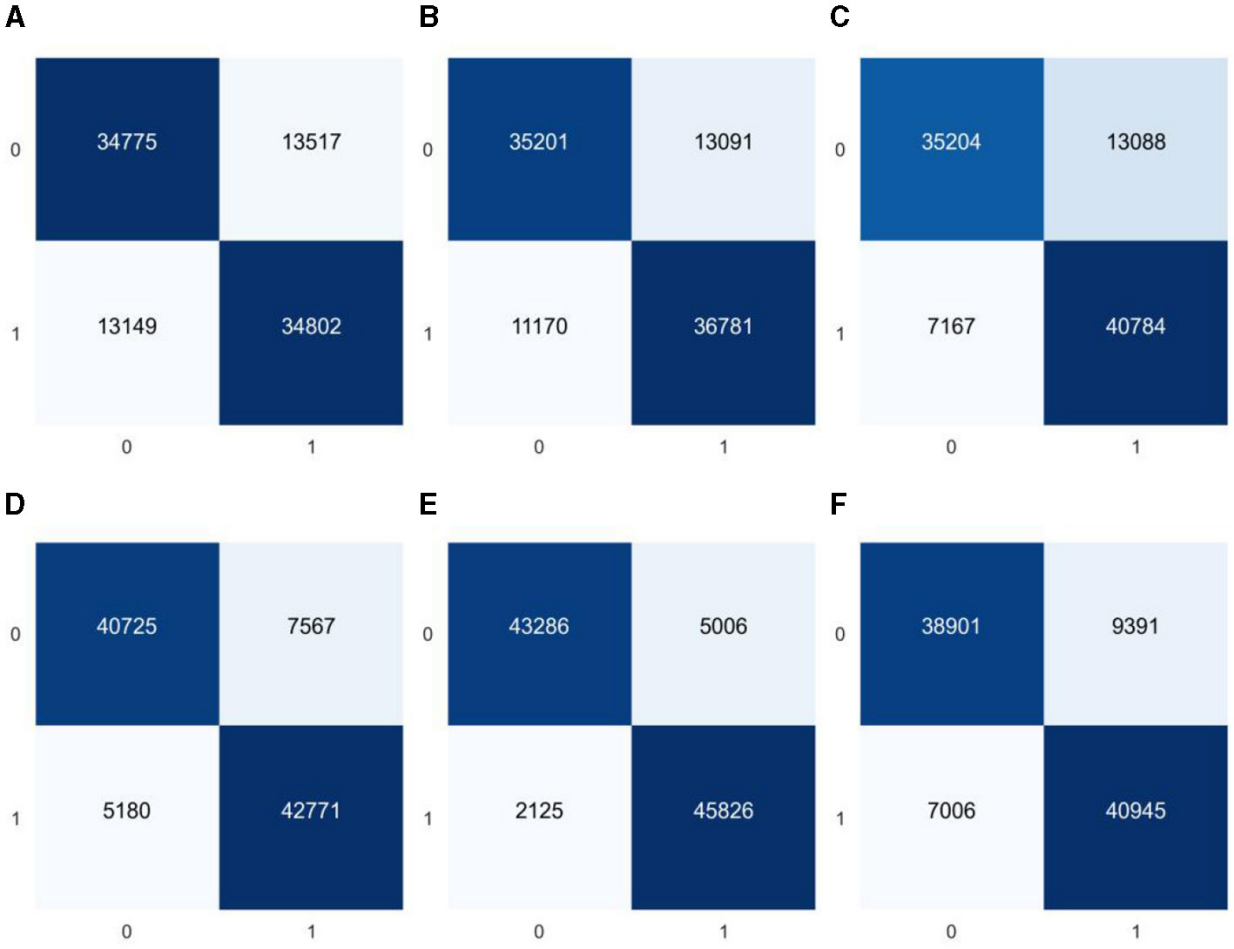
Classification confusion matrices for predictions of six common machine learning models based on balanced datasets **(A)** GaussianNB, **(B)** LR, **(C)** KNN, **(D)** CART, **(E)** RF, and **(F)** GBDT.

In the equation, *x*_*i*_ represents the feature vector corresponding to the i-th sample in the minority class; *x*_*n*_ represents the new feature vector synthesized based on *x*_*i*_; *x*_*k*_ represents a randomly selected sample vector from the nearest neighbor vectors of *x*_*i*_; and ζ represents a uniform random variable between 0 and 1. The newly generated sample variables are composed of finite decimals, so all feature variables except for BMI are rounded to integers to make their values more realistic. The experiment found that the dataset was more ideal when the number of nearest neighbor vectors was 7, and the balanced dataset, after numerical correlation tests, was found to be more consistent with the influence of feature variables on diabetes in the unbalanced dataset. To minimize the impact of duplicate values on the accuracy of the dataset, this article underwent five rounds of balancing to ensure that it did not contain any duplicate values. The confusion matrix of the classification, tested using multiple ML algorithms, is shown in Figure 5. By analyzing the confusion matrices before and after equalization, a reduction in variability between categories can be clearly observed. The balanced confusion matrix shows a more balanced distribution of true positives and true negatives, especially the model's performance in identifying diabetes samples (category 1) has been significantly improved. This shows that data balancing has a positive impact on improving the model's ability to identify the minority class (people with diabetes).

This study uses accuracy and F1 score as metrics to evaluate model performance. The accuracy rate is the ratio of the number of samples correctly predicted by the model to the total number of samples. It is an intuitive indicator to measure whether the model prediction is correct. The F1 score is the harmonic mean of precision and recall, which provides a more balanced assessment of model performance, with higher values indicating better performance. In addition, considering the algorithm running time cost of enumerating feature variables, this study also takes the time spent on model training and construction as one of the evaluation criteria. To obtain better prediction results, during the parameter selection process, we first set the initial parameters based on the theoretical basis of each algorithm and experience in previous literature to ensure that the selected parameters can maintain good stability on the test set and accuracy. In terms of fine-tuning, we adjusted the core parameters of the algorithm, such as the regularization term, kernel function type (for SVM), number of neighbors (for KNN), tree depth and number of leaves (for decision trees and tree-based ensemble algorithms), and learning rate. These fine tunings are designed to optimize the model's performance on a specific data set while preventing overfitting and ensuring that the model has good generalization capabilities. Finally, the best set of prediction results are obtained, as shown in Table 5.

**Table 5:** Performance comparison of various machine learning (ML) models.

**Algorithmic model**	**Accuracy (Unit: %)**	**F1 (Unit: %)**	**Calculation time (Unit: s)**
GaussianNB	72.31	72.35	0.22
LR	75.04	75.52	0.31
SVM	76.67	77.74	6,406.89
KNN	79.07	80.26	45.93
CART	86.99	87.24	2.07
Bagging	90.75	91.1	247.77
RF	92.47	90.46	73.51
GBDT	83.05	83.43	36.93
XGBoost	90.64	90.19	14.53
LightGBM	90.26	89.72	1.39

Due to the balanced processing of the dataset, the model's classification accuracy and F1 score (the combined effect of precision and recall) will show relatively consistent results on the test set. As shown in Table 5, the Bagging, RF, XGBoost, and LightGBM algorithms performed well in terms of accuracy and F1 on the test set, indicating that the ensemble learning approach performed well in handling these one-dimensional vector data with multiple variables. The bagging and random forest algorithms are based on the bagging idea in the ensemble learning framework, using data sampling to build multiple base classifiers for parallel computation. By integrating the predictions of multiple decision trees to obtain the final prediction result, the variance of the model can be reduced. For the dataset in this study, the random forest used as the base classifier for the bagging algorithm yielded the best results. However, implementing the bagging idea requires training multiple decision trees, and each decision tree needs to process a portion of the training data, resulting in a higher computational cost for datasets with a large sample size and a high number of features, as demonstrated by the computational time in the table. This does not meet the needs of feature enumeration in this study.

The XGBoost and LightGBM algorithms are optimizations of the GBDT algorithm, which is an ensemble learning technique based on decision trees. These algorithms utilize the boosting method in the ensemble learning framework, which involves using a series of base classifiers to continuously improve and ultimately create strong classifiers for ensemble learning. The core principle of GBDT, XGBoost, and LightGBM is to gradually reduce the loss function using gradient descent to approach the true value of the predicted result. Their prediction functions can be expressed as the sum of the predicted values from multiple trees, and they all incorporate a learning rate to control the contribution of each tree. Equation 5 can be used to represent their core formulas.


(5) $$\begin{array}{l} {\text{F}}_{\text{m}}\left(\text{x}\right)={\text{F}}_{\text{m}-1}\left(\text{x}\right)+\eta {\text{h}}_{\text{m}}\left(\text{x}\right) \end{array}$$

Where *F*_*m*−1_(*x*) represents the sum of predicted values from the first m-1 trees; *h*_*m*_(*x*) represents the predicted value of the m-th tree; η represents the learning rate which controls the contribution of each tree. By iteratively training multiple trees, the final prediction function F(x) can be represented by Equation 6:


(6) $$\begin{array}{l} \text{F}\left(\text{x}\right)=\overset{\text{M}}{\underset{\text{m}=0}{\sum }}\eta {\text{h}}_{\text{m}}\left(\text{x}\right) \end{array}$$

In the equation: *M* represents the number of trees. When the decision tree depth is too large, the model is overly matched to the training data, and GBDT is prone to overfitting, which reduces the model's generalization ability. This can be seen from Table 5, where the accuracy of the GBDT model is 7 percentage points lower than that of the XGBoost and LightGBM models. XGBoost addresses this issue by adding L1 or L2 regularization terms to the objective function and using techniques such as feature subsampling and row sampling to effectively reduce the risk of overfitting and improve the model's generalization ability (32). For the dataset in this study, experiments show that using L1 regularization improves the model's performance. This allows some feature weights to be zero, achieving the effect of feature selection. The objective function is represented by Equation 7:


(7) $$\begin{array}{l} {\text{obj}}^{\left(\text{t}\right)}=\overset{\text{n}}{\underset{\text{i}=1}{\sum }}\text{l}\left({\text{y}}_{\text{i}}{{,\hat{\text{y}}}_{\text{i}}}^{\left(\text{t}-1\right)}+{\text{f}}_{\text{t}}\left({\text{x}}_{\text{i}}\right)\right)+\lambda_{1}\overset{\text{K}}{\underset{\text{j}=1}{\sum }}|\omega_{\text{j}}| \end{array}$$

In the equation: *t* represents the current iteration number; *n* represents the number of samples; *l* represents the loss function; *y*_*i*_ represents the true label of the i-th sample; ${{ŷ}_{i}}^{\left(t-1\right)}$ represents the predicted value of the model for sample i after t-1 rounds of iteration; *f*_*t*_(*x*_*i*_) represents the predicted value of the t-th base learner for the i-th sample; λ_1_ represents the L1 regularization coefficient, the larger its value, the stronger the regularization, and the smaller the complexity of the model; *K* represents the number of leaf nodes; ω_*j*_ represents the output of the j-th leaf node. The LightGBM algorithm used in this article also uses L1 regularization to achieve the best classification performance. In terms of data sampling, LightGBM uses gradient-based one-side sampling [36], which only samples the part of data with larger sample gradients during the construction process of each decision tree, reducing computational complexity and improving performance.

Based on the analysis of algorithm computation time, gaussian naive Bayes and logistic regression algorithms have low computation costs since they don't require extensive adjustment of feature values. However, they may exhibit underfitting when confronted with multiple feature dimensions and large data volume. On the other hand, the training process of SVM algorithm involves a significant number of matrix operations and optimization calculations, resulting in high computation costs for the large dataset used in this article. As indicated in Table 5, its computation time cost exceeds that of other algorithms by far. The KNN algorithm needs to compute the distance between the target sample and all training samples, and this computation cost escalates rapidly with dataset size, causing high computational complexity and impacting algorithm efficiency. Conversely, Boosting-based algorithms have demonstrated their advantage in terms of runtime. XGBoost utilizes the data block structure parallel split and search, along with cache-aware prefetching algorithms, to improve algorithm efficiency (32). LightGBM continues to use the histogram algorithm to discretize continuous features on the selection of optimal split points, thereby converting continuous features that require sorting into discrete feature values, significantly reducing memory consumption. It also applies gradient-based one-side sampling and exclusive feature bundling techniques (33) to reduce the number of data samples that require computation. Table 5 data shows that LightGBM has greatly improved its computational efficiency for large data compared to its original GBDT algorithm.

To clearly understand the effectiveness of data standardization measures, comparative experiments were also conducted using 6 representative machine learning algorithms under the same conditions. The experimental results in the Table 6 show that after applying data standardization, the accuracy and F1 score of multiple algorithm models have been improved to a certain extent, and the convergence speed of the algorithm has been greatly accelerated. For example, the calculation time of the random forest model was reduced by about 17s. Experiments show that using data normalization does improve the performance and convergence speed of the model.

**Table 6:** Performance comparison of 6 algorithms before and after using data standardization.

**Algorithmic model**		**Accuracy (Unit: %)**	**F1 (Unit: %)**	**Calculation time (Unit: s)**
GaussianNB	Not standardized	72.23	72.11	0.22
	Standardization	72.31	72.35	0.22
LR	Not standardized	70.72	71.98	2.93
	Standardization	75.04	75.52	0.31
KNN	Not standardized	78.88	80.08	58.24
	Standardization	79.07	80.26	45.93
CART	Not standardized	86.81	87.05	2.53
	Standardization	86.99	87.24	2.07
RF	Not standardized	92.41	90.21	90.48
	Standardization	92.47	90.46	73.51
LightGBM	Not standardized	83.02	82.03	46.25
	Standardization	83.05	83.43	36.93

When choosing the most suitable model, this study considers the trade-off between accuracy and execution efficiency. For application scenarios that require fast processing, LightGBM may be a better choice, although some accuracy is sacrificed. In cases where higher accuracy is required, RF may be a more suitable choice, although it requires longer calculation time. Considering that the subsequent enumeration experiments of this study require high computational efficiency of the algorithm, the LightGBM algorithm was selected to be used in subsequent experiments to study the impact of feature vector combinations on model prediction performance.

### 3.3 Comparison of equilibrium methods

To optimize the recall rate of the machine learning model for the people with diabetes, this study further compared a variety of advanced oversampling techniques. These techniques include K-Nearest Neighbors Over-sampling (KNNOR) and several variants of SMOTE, which are comprehensively compared with the original SMOTE method to evaluate their performance in data set balancing. The KNNOR method uses the K nearest neighbor algorithm to generate new synthetic samples for the area around minority class samples. The core of this method is to give priority to minority class samples that are difficult to classify correctly on the classification boundary. KNNOR provides a more efficient way to handle highly imbalanced data sets by defining the neighborhoods of minority class samples more accurately.

In addition to the standard SMOTE method, there are also various SMOTE variants, such as Borderline-SMOTE, KMeansSMOTE, and SVMSMOTE. The Borderline-SMOTE method focuses on processing minority class samples that are close to the majority class boundary, identifying these boundary samples and generating new samples around them to enhance the model's ability to discriminate boundary areas. KMeansSMOTE combines K-means clustering and SMOTE technology. By clustering minority class samples and then applying the SMOTE method in each cluster, it can cover the distribution of minority class samples more evenly. SVMSMOTE uses support vector machines (SVM) to identify samples that are difficult to classify, and then applies the SMOTE algorithm in the neighborhoods of these samples to enhance the model's learning effect on these difficult samples. This study applies SMOTE, KNNOR, Borderline-SMOTE, KMeansSMOTE and SVMSMOTE technologies to balance the diabetes data set, and uses the excellent LightGBM algorithm to test the effect. To ensure the authenticity and validity of the data, we only retain unique samples after each equalization process and exclude duplicate values. The experimental results, shown in Table 7, provide a performance comparison of these techniques on balanced datasets.

**Table 7:** Comparison of the effects of 5 equalization methods based on LightGBM model.

**Balancing method**	**Accuracy (Unit: %)**	**F1 (Unit: %)**	**Balanced dataset time (Unit: s)**
SMOTE	88.56	86.66	3.94
KNNOR	83.89	81.82	95.37
Borderline-SMOTE	88.23	86.05	21.64
KMeansSMOTE	90.31	89.07	10.02
SVMSMOTE	88.83	86.7	2,047.24

In this study, we compare the performance of five oversampling methods, SMOTE, KNNOR, Borderline-SMOTE, KMeansSMOTE, and SVMSMOTE, in balancing diabetes datasets. Metrics we focus on include accuracy, F1 score, and time required for data set balancing. Combining these indicators, KMeansSMOTE performed the most outstandingly among all methods, leading with an accuracy of 90.31% and an F1 score of 89.07%. At the same time, it only took 10.02 seconds to complete data balancing, showing high efficiency and effectiveness. Although SVMSMOTE performs well in terms of accuracy and F1 score, the time required (2,047.24 seconds) is too long, which limits its practical application. On the other hand, although SMOTE and Borderline-SMOTE process faster, they are not as good as KMeansSMOTE in performance. KNNOR performs the worst in terms of accuracy and F1 score, possibly due to its limitations in dealing with extremely imbalanced data sets. For this research data set, KMeansSMOTE is an efficient and effective choice to improve the recall rate and overall classification performance of the diabetes data set, and this study believes that KMeansSMOTE is particularly suitable for application scenarios that require a balance between processing speed and accuracy.

### 3.4 Feature variable enumeration

**Figure 6:**
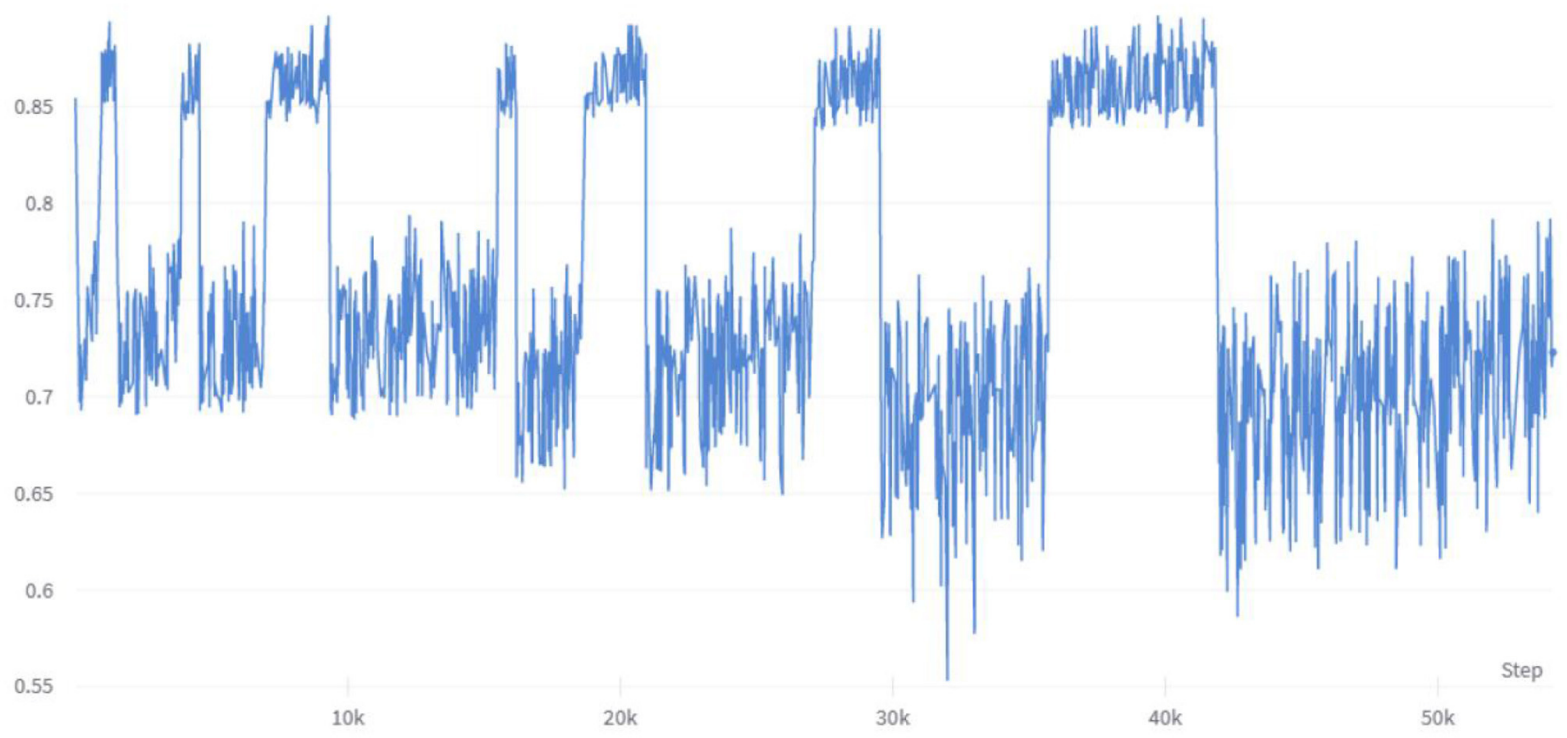
Changes in accuracy under enumeration of 6 feature variables.

To investigate the impact of multiple combinations of diabetes risk factors on diabetes risk and the correlation between each feature vector and its impact on risk prediction, this study employed a combination of enumerating feature variable combinations with the LightGBM algorithm. The accuracy of the model was used to determine the strength of the impact of feature variable combinations on diabetes risk prediction, and to verify the ranking of individual feature variables in relation to their correlation with diabetes. Initially, six feature variable combinations were selected from the 21 available, resulting in a total of 54,264 combination methods, with their corresponding accuracy changes shown in Figure 6. The figure indicates that different feature variable combinations can cause a range of approximately 35 percentage points in model prediction accuracy, suggesting that different feature variable combinations can have a significant impact on the performance of diabetes risk prediction models. The waveform in the figure shows a high accuracy waveform change at regular intervals, indicating that these diabetes feature combinations contain feature variables that have a strong correlation with diabetes risk. This not only demonstrates the feasibility of the approach proposed in this article to use the efficient LightGBM algorithm to enumerate feature variables to explore the strength of their impact on diabetes, but the peak values in the figure also indicate that this method can indeed be used to simplify the number of feature variables in risk questionnaires.

**Figure 7:**
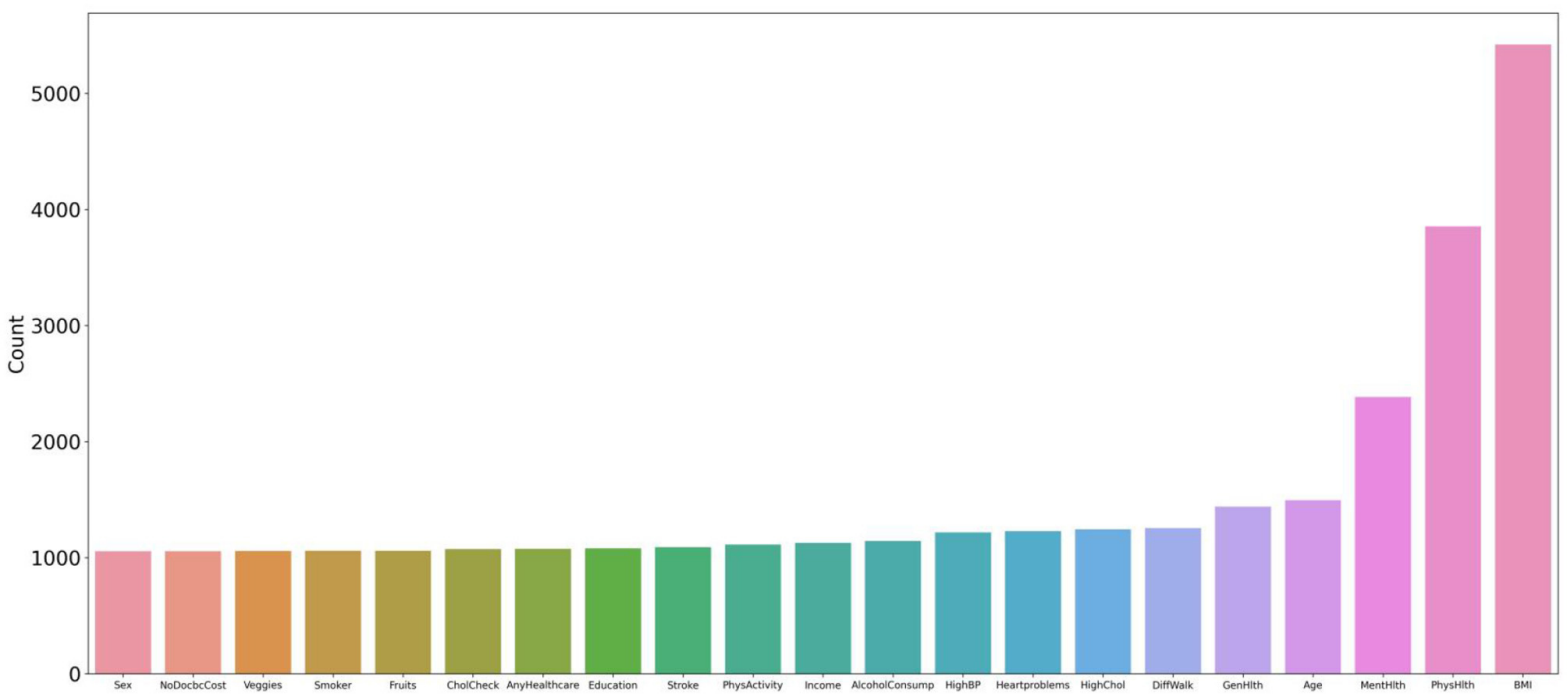
Statistics and ranking of occurrences of feature variables. The figure shows that the characteristic variables with higher frequency include BMI, PhysHlth, MentHlth, Age, and GenHlth.

The analysis of the experimental data of the six feature variables shows that the combination of HighChol, BMI, GenHlth, MentHlth, PhysHlth, and Age has the highest accuracy, reaching 89.89 %, which is close to the accuracy of the full variable of 90.26 %. This indicates that the feature variables with strong diabetes correlation are in this variable combination, and the feature variable combination with a simplified variable number of 6 can still achieve higher accuracy. To test the strength of correlation between feature variables and diabetes risk prediction, this study extracted all feature combinations represented by accuracy values in the top 10% based on generated experimental data, and counted the total number of occurrences of each feature variable. Figure 7 shows the occurrence frequency and ranking of the feature variables. From the figure, it is evident that the feature variables with higher occurrence frequency include BMI, PhysHlth, MentHlth, Age, and GenHlth. This finding suggests that obesity, poor mental health, and advanced age are the most relevant risk factors for judging whether a person suffers from diabetes when six variables are enumerated to generate multiple combinations. It also suggests that the more frequent the occurrence of physical and mental discomfort in daily life, the higher the likelihood of developing diabetes. Based on multiple experiments, this study also found that the higher the accuracy of the combination of feature variables, the gap between the occurrence frequency of these highly correlated feature factors and other feature will increase, which means that they have a stronger impact on the prediction of diabetes risk.

## 4 Discussion

**Figure 8:**
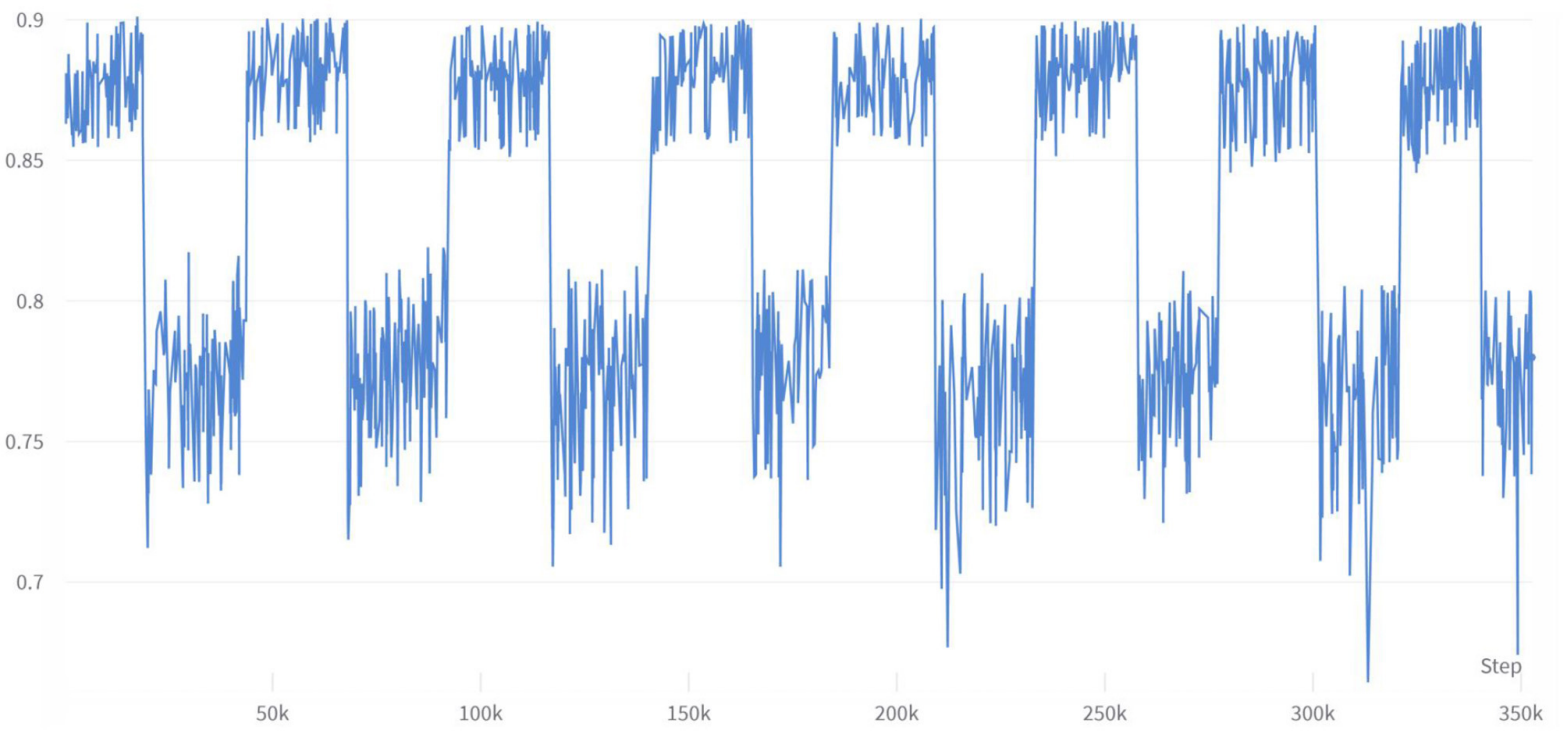
Changes in accuracy under enumeration of 11 feature variables.

This study conducted multiple enumeration experiments with various feature variables, and the accuracy waveform obtained was consistent with the trend in Figure 6. Furthermore, the lower limit of accuracy in the waveform increases as the number of variables increases, while the upper limit approaches the accuracy of the full-variable model. This trend change can be clearly seen in Figure 8. The model's performance almost reached that of the full-variable model after increasing the number of feature variables from 6 to 11, and further increasing the number of variables had little effect on the model's generalization performance. After counting the number of feature variables with higher accuracy included in the statistical experimental data, Table 8 shows the ranking and proportion of the correlation between the optimized feature variables in the dataset and diabetes. According to the experimental data, obesity is the most significant risk factor affecting the incidence of diabetes, and its impact is much greater than that of other risk factors. Therefore, people with obesity should pay extra attention to their own risk of diabetes. Age, high cholesterol, high blood pressure, coronary heart disease, or myocardial infarction are also high-risk factors for diabetes. On the other hand, factors such as smoking, gender, vegetables, and fruits have a weak correlation with diabetes and relatively little impact on the performance of the diabetes risk model. Therefore, these factors can be removed from the diabetes survey questionnaire to improve the efficiency of data collection without affecting the overall performance of the model. The experiment also found that people with lower income are at a higher risk of developing diabetes, indicating that they need to be more concerned about diabetes health, and the theoretical mechanisms underlying their indirect influence require more extensive social research.

**Table 8:** Feature variables ranking for diabetes risk relevance.

**Ranking**	**Feature variables**	**Proportion**	**Risk meaning**
1	BMI	17.69%	Obesity
2	PhysHlth	6.56%	Poor Physical health days/month
3	MentHlth	6.56%	Poor mental health days/month
4	Age	5.75%	advanced age
5	GenHlth	4.87%	Self-health perception
6	HighChol	4.33%	High cholesterol
7	HighBP	4.30%	High blood pressure
8	AlcoholConsump	4.14%	Alcoholism
9	Heartproblems	4.02%	Coronary heart disease or myocardial infarction
10	DiffWalk	3.94%	Difficulty in walking
11	Income	3.76%	Low family income
12	PhysActivity	3.48%	Lack of physical activity
13	Stroke	3.46%	Stroke
14	Education	3.44%	Low education
15	CholCheck	3.42%	No cholesterol checking
16	Smoker	3.40%	Smoker
17	Sex	3.39%	Sex
18	Veggies	3.38%	No habit of eating veggies every day
19	AnyHealthcare	3.38%	No health insurance
20	Fruits	3.37%	No habit of eating fruit every day
21	NoDocbcCost	3.36%	No money to see a doctor

The performance of the hardware equipment used in this research is not strong enough, so only 21 feature variables are selected to carry out the enumeration feature experiment combined with the efficient LightGBM algorithm. If you face a data set with a large number of data samples and increase the number of data features, the time cost of the experiment will be greatly increased, and the requirements for hardware computing performance will be higher.

SMOTE is used in this study to solve the problem of unbalanced number of data samples. Although the balanced data set has been tested to show that the magnitude of the numerical correlation of the character variables on diabetes does not cause large changes, the synthetic samples generated by the SMOTE are created by interpolating the original samples, which may lead to some unreal samples appear. These synthetic samples may not fully reflect the distribution of real samples, thus introducing some bias. We will conduct further optimization research on this to reduce the synthesis error. In the future, we also will hope to consider threshold shifting and category weighting methods to increase the recall ability of the model for minority categories.

## 5 Conclusion

Based on 2021 BRFSS survey data, this study utilized the efficient LightGBM algorithm and enumerated feature variables to demonstrate the correlation ranking of various risk factors with the risk of diabetes. The results show that obesity has the strongest impact on the risk of diabetes, far exceeding other risk factors. In addition, psychological factors, advanced age, high cholesterol, high blood pressure, alcohol abuse, coronary heart disease or myocardial infarction, mobility difficulties, and low family income are also correlated with the risk of diabetes to some extent. The experimental data in this study demonstrate that, while maintaining a comparable level of accuracy, the questionnaire variables and the number of questions can be significantly optimized, making follow-up more efficient and better suited for precise diabetes prevention. Furthermore, the research methods employed in this study have certain reference value for studying the risk correlation of other diseases, and the research results help to increase society's attention to populations at greater risk of diabetes. This is especially important in the context of the current COVID-19 pandemic, where the construction of diabetes risk models and the problem of precise disease prevention should be given more attention.

## Data availability statement

The original contributions presented in the study are included in the article/supplementary material, further inquiries can be directed to the corresponding author.

## Author contributions

LJ: Conceptualization, Methodology, Project administration, Writing—review & editing. ZY: Conceptualization, Data curation, Methodology, Software, Supervision, Writing—original draft. GL: Methodology, Writing—review & editing. ZX: Software, Writing—review & editing. GY: Software, Writing—review & editing. HG: Investigation, Validation, Writing—review & editing. JW: Investigation, Validation, Writing—review & editing. LW: Funding acquisition, Supervision, Writing—review & editing.

## Footnotes

^1^Available online at: https://www.kaggle.com/datasets/andrewmvd/early-diabetes-classification.

^2^Available online at: https://www.cdc.gov/brfss/annual_data/annual_2021.html.
